# A Framework to Analyze Primate Species Vulnerability to Trade in Urban Markets

**DOI:** 10.1002/ajp.70102

**Published:** 2025-12-07

**Authors:** Christian J. Rivera, Jessica S. Kahler, Wahyu Nurbandi, Agustín Fuentes

**Affiliations:** ^1^ School for the Environment University of Massachusetts Boston Boston Massachusetts USA; ^2^ High Meadows Environmental Institute Princeton University Princeton New Jersey USA; ^3^ Department of Anthropology Princeton University Princeton New Jersey USA; ^4^ Department of Sociology and Criminology & Law University of Florida Gainesville Florida USA; ^5^ Princeton School of Public and International Affairs Princeton University Princeton New Jersey USA

**Keywords:** green criminology, hunting, poaching, target suitability framework, wildlife trade, wildlife trafficking, caza, caza furtiva, comercio de vida silvestre, criminología verde, marco de idoneidad del objetivo, tráfico de vida silvestre

## Abstract

Live primates are increasingly commodified and appropriated as inputs into markets, both at the international and domestic scales, and across physical and online platforms. We present a qualitative and quantitative framework that integrates concepts in primatology and conservation criminology toward understanding the characteristics that make different primate species vulnerable to trade in domestic urban markets. Our market‐stage and live animal‐focused framework relates primate characteristics to both supply‐ (i.e., opportunity‐based) and demand‐side (i.e., consumer‐driven) variables. Supply‐side variables include *concealability*, *abundance*, *accessibility*, and *removability*, while demand‐side variables include *processability*, *replaceability*, *usability*, *enjoyability*, and *value*. We empirically test our framework using representative data from a specific urban market and time period in Indonesia (Medan, Sumatra; 1997–2008) to illustrate its utility in explaining why more individuals of certain species are detected in markets over others, and to elucidate the roles of supply‐ and demand‐side drivers of trade within the focal context. Results from a principal component analysis show that both supply‐ (high abundance, accessibility, and concealability) and demand‐side variables (high usability/ecological value, low rarity) explained the high detection of macaques and lorises in the wildlife markets of Medan during 1997–2008 when compared to the lower numbers of leaf monkey and gibbon species. This primate‐focused conservation criminology framework is flexible and can be adapted to examine live primates in legal and illicit trade across other primate‐range countries and scales, and in contexts beyond physical urban markets such as online fora.

## Summary

- We present a qualitative and quantitative conservation criminology framework to analyze primate taxon‐specific vulnerability to live trade in urban markets.
- The framework is both explanatory and predictive and relates primate characteristics to supply‐ (i.e., opportunity‐based) and demand‐side (i.e., consumer‐driven) variables.
- This new tool can be used to examine primates in trade across diverse physical and online markets toward informing the development of supply‐ and/or demand‐side interventions to mitigate live primate trade.

## Graphical abstract

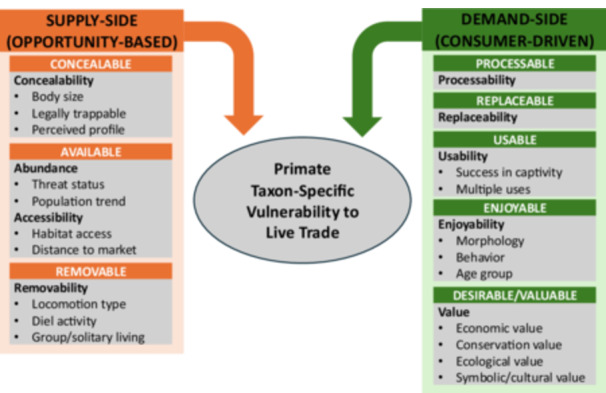

## Resumen

Los primates vivos son cada vez más comercializados y apropiados como insumos para los mercados, tanto a escala internacional como nacional y en plataformas físicas y en línea. Presentamos un marco cualitativo y cuantitativo que integra conceptos de primatología y criminología de la conservación para comprender las características que hacen que diferentes especies de primates sean vulnerables al comercio en los mercados urbanos domésticos. Nuestro marco, centrado en la etapa de comercio y en los animales vivos, relaciona las características de los primates con variables tanto del lado de la oferta (es decir, basadas en la oportunidad de caza y venta) como del lado de la demanda (es decir, impulsadas por el consumidor). Las variables del lado de la oferta incluyen las características de *ocultable*, *abundante*, *accesible* y *removible*, mientras que las variables del lado de la demanda incluyen *procesabilidad*, *reemplazable*, *útil*, *agradable* y *valor*. Probamos empíricamente nuestro marco usando datos representativos de un mercado urbano en un período de tiempo específico en Indonesia (Medan, Sumatra; 1997–2008) para ilustrar su utilidad para explicar por qué se detectan más individuos de ciertas especies en los mercados que de otras, y para dilucidar los roles de los impulsores de oferta y demanda del comercio dentro del contexto focal. Los resultados de un análisis de componentes principales muestran que tanto las variables de oferta (alta abundancia, accesibilidad y ocultabilidad) como las de la demanda (alta utilidad/valor ecológico, baja rareza) explicaron la alta detección de macacos y loris en los mercados de vida silvestre de Medan durante 1997‐2008 en comparación con los números más bajos de especies de langurs y gibones. Este marco de criminología de la conservación es flexible y se puede adaptar para examinar primates vivos en el comercio legal e ilícito en otras escalas y países de distribución de primates, y en contextos más allá de los mercados urbanos físicos, como los foros en línea.

**Abbreviations**
CAPTURED: concealable, available, processable, transferable, useable, removable, enjoyable, and desirable
CRAVED: concealable, removable, available, valuable, enjoyable, and disposable
IUCN: International Union for the Conservation of Nature

## 1 Introduction

More than half of the world's primate species are threatened with extinction and about 75% of species have populations in decline (Estrada et al. 2017). The impending extinction crisis of the world's primates is driven primarily by anthropogenic activities, including unsustainable hunting, trapping, and trade of primates across their range (Estrada et al. 2017, 2018; Alexander et al. 2023). While primates are harvested and consumed for subsistence purposes, live individuals and body parts are also commercially traded to meet demand in biomedical research, the exotic pet trade, traditional medicine, and ornamental use, among others (Estrada et al. 2017; Blair, Le, Sterling, et al. 2017; Gamalo et al. 2024; Badihi et al. 2024). The magnitude and impacts of the primate trade on species and populations have received growing interest among primatologists and conservationists, as illustrated by journal special issues (e.g., Nijman et al. 2011; Blair, Le, Sterling, et al. 2017), yet relatively little is known about the domestic trade in live primates within their range countries (Badihi et al. 2024), particularly the drivers of trade. There is a growing demand for live primates by urban consumers throughout range countries, with concerns that opportunistic trapping of live primates is shifting to more organized trafficking networks (Linder et al. 2013; Nijman et al. 2017, 2023; Norconk et al. 2020; Gamalo et al. 2024; Garber et al. 2024).

It is difficult to calculate the exact numbers of live primates traded per year. Nevertheless, there is strong evidence and widespread consensus among primatologists and conservationists that the live primate trade, particularly to supply the pet trade, is increasing (Norconk et al. 2020; Alexander et al. 2023). Conservative estimates on international trade (i.e., cross‐border trade) suggest that over 450,000 live primates and over 11,000 total individuals in the form of body parts were traded legally and illegally in the time period 2005–2014 (Estrada et al. 2017). Most of the individuals (93%) destined for international markets were Asian primates (Estrada et al. 2017). The United States, China, and Japan are the largest importers of live primates, primarily to meet demand for biomedical research (Nijman et al. 2011; Linder et al. 2013). While we are increasingly learning about the drivers of the primate trade at the international level, a significant gap exists in our understanding of the complex social‐ecological factors that drive commercial trade in primates at the domestic scale (Blair, Le, Thạch, et al. 2017; Rivera et al. 2021; Alexander et al. 2023). Moreover, one of the major research gaps in studies of primate conservation is understanding how primate species‐specific characteristics relate to and interact with human social factors to hinder or drive the hunting and trade of primates by humans across diverse local and domestic contexts (Blair, Le, Sterling, et al. 2017; Blair, Le, Thạch, et al. 2017; Rivera et al. 2021).

In light of these concerns, primatologists and crime science scholars call for studies that develop and operationalize criminological frameworks toward disentangling the complex and diverse drivers of the primate trade (Estrada et al. 2017; Wilson and Kurland 2019; Rivera et al. 2021). The growing field of conservation criminology draws on approaches from conservation science, criminology, and risk and decision sciences to address issues of environmentally relevant crimes, noncompliance, and risks (Gibbs et al. 2010; Kahler et al. 2021; Gore and Bennett 2022). This interdisciplinary field offers new insights for the development of studies on drivers of wildlife trade. Various frameworks originating in criminology to examine property theft and predatory crimes have been adapted to examine the characteristics of “hot products” (species) that are disproportionately targeted for theft (poaching) and trade; these are referred to as “target‐suitability frameworks” (see Kahler et al. 2022 for a discussion of the evolution of target suitability frameworks in conservation criminology). The CRAVED framework, for example, has been used to examine the concealable, removable, available, valuable, enjoyable, and disposable characteristics of poached animals such as parrots in Bolivia, Mexico, and Indonesia (Pires and Clarke 2012; Pires and Petrossian 2016; Pires et al. 2021) and fish species targeted by commercial fishers (Petrossian and Clarke 2014). The operationalization of the CRAVED framework has helped to elucidate the supply‐ (i.e., opportunity‐based) and demand‐side (i.e., consumer‐driven) factors influencing the poaching of different wildlife species.

The aforementioned studies on the application of target‐suitability frameworks focused mainly on specific actors (e.g., commercial harvesters or vendors) or supply chain stages (e.g., poaching stage). Moreto and Lemieux (2015) extended the CRAVED framework and introduced CAPTURED, a market‐stage, product‐focused framework to examine the concealable, available, processable, transferable, useable, removable, enjoyable, and desirable/valuable characteristics of wildlife products. The framework serves as a lens to examine how the specific characteristics of the traded products or species affect their propensity to be poached and sold in illicit markets (Moreto and Lemieux 2015). The CAPTURED framework has recently been applied to qualitatively analyze the convergence of illegal fishing and human trafficking (Moreto et al. 2020). Ample opportunities exist to operationalize the CAPTURED and other target‐suitability frameworks and assess their utility in understanding why certain species are more vulnerable to trade than others, including assessing the roles of supply‐ and demand‐side factors in driving trade within specific social‐ecological contexts (Moreto and Lemieux 2015).

Given growing concerns regarding the trade of live primates in urban markets globally (Norconk et al. 2020; Alexander et al. 2023; Gamalo et al. 2024; Badihi et al. 2024), the main goals of this study are threefold. First, to advance the fields of primatology and conservation criminology by integrating considerations of primate‐relevant biological, ecological, and conservation science‐based dimensions within a market‐stage and wildlife product‐focused target‐suitability framework. Second, to provide a framework for both qualitative and quantitative assessments of (1) primate species vulnerability to live trade and (2) supply‐ and demand‐side drivers of trade. Third, to illustrate the utility of the proposed framework by applying it to representative market data. While we empirically test our framework using data from Indonesia within a specific urban market and time period, it can be adapted to examine live primates in trade across other primate‐range countries, contexts, and scales.

## 2 Description

**Figure 1:**
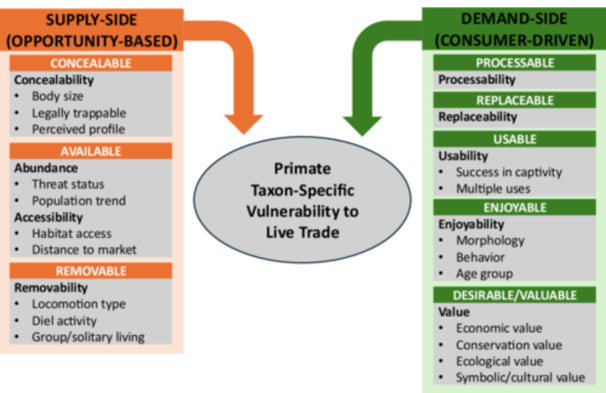
A primate‐focused conservation criminology framework to examine taxon‐specific vulnerability to live trade in urban markets.

We propose a framework to analyze primate species vulnerability to live trade, drawing primarily on the conservation criminology CAPTURED target‐suitability framework to analyze illicit trade of wildlife products (Figure 1 and Table 1) (Moreto and Lemieux 2015). The CAPTURED framework proposes that wildlife products that are concealable, available, removable, processable, transferable/disposable, usable, enjoyable, and desirable/valuable are more prone to theft and illicit trade (Moreto and Lemieux 2015). These product characteristics can be categorized as either supply‐ (opportunity‐based) or demand‐side (consumer‐driven) variables. To inform the selection of relevant variables for analysis of live primate trade, we drew on literature on primate ecology and conservation (e.g., Galán‐Acedo et al. 2019) and studies on development and applications of target‐suitability frameworks to other taxa (Pires and Clarke 2012; Petrossian and Clarke 2014; Pires 2015; Pires and Petrossian 2016; Pires et al. 2021; Kahler et al. 2022). We note that our list of variables is not exhaustive.

**Table 1:** Framework and variables for analysis of primate species vulnerability to live trade.

Conservation criminology element	Theme	Variable	Variable description/assumptions
*Supply‐side (opportunity‐based)*			
Concealable	Concealability	Body size	Body size may affect how difficult it is to hide a species (e.g., in transport, within stalls in an open market)
		Legally trappable	Species legally trappable up to certain quotas may be more concealable than those fully protected by law
		Perceived profile	Low‐profile species may attract less attention from authorities than high‐profile species; the perceived profile of the species may or may not be related to its legal protection
Available	Abundance	Threat status	Conservation/threat status; how common the species is in the wild or in the focal site (population density)
		Population trend	The population trend of the species: for example, decreasing, stable, increasing
	Accessibility	Habitat access	Some habitats may be easier or more difficult to access, for example, anthropogenic habitats such as farms may be easier to access; increased access by humans through habitat encroachment (infrastructure development, mining, plantations)
		Distance to market	Species that inhabit a catchment area closer to the market may be more vulnerable to trapping and trade
Removable	Removability	Locomotion type	Main form of movement in its environment: primarily arboreal, terrestrial, or commonly active on both ground and in trees; arboreal primates may be more difficult to capture than terrestrial ones, but this may be affected by technology (e.g., guns)
		Diel activity	Primarily active during the day (diurnal), night (nocturnal), or both (cathemeral); nocturnal species may be more evasive than diurnal species
		Group/solitary living	Group size; social cohesion; primates living in groups may increase chances of at least one individual being captured
*Demand‐side (consumer‐driven)*			
Processable	Processability	Processability	Ability of individual live primate to be altered or modified, for example, teeth removal to modify aggressive behavior or give appearance of modified behavior
Replaceable (transferable/disposable)	Replaceability	Replaceability	Found in multiple markets; easily replaced if given away or if it dies; easy to move between markets
Usable	Usability	Success in captivity	Ecological and behavioral flexibility; diet flexibility (e.g., restricted diet vs. high diet flexibility); tendency to have high or low mortality in captive environments; risk of disease or infection
		Multiple uses	Multiple uses after being purchased live, for example, pet, protein source, pest control, medicine, entertainment, income generation
Enjoyable	Enjoyability	Morphology	Primates that are smaller in size are known to be prominent in the domestic trade due to their enjoyability as small pets that can be carried; visually appealing
		Behavior	Individuals with tamer behaviors may be more enjoyable; some species may be trained (e.g., to perform)
		Age group	Younger individuals, particularly those who have not reached sexual maturity, may be perceived as more enjoyable
Desirable/valuable	Value	Economic value	High monetary value may indicate consumer demand
		Conservation value (i.e., rarity)	Species rare in the wild or endemic to a certain site may be more desirable
		Ecological value	Species may hold ecological value to humans beyond co‐sharing space as pets/companions (e.g., pest control, derivatives for protein or medicine)
		Symbolic/cultural value	Desirable to own due to its symbolic values and meanings (e.g., culture, social status)

*Note:* Conservation criminology elements are derived from target‐suitability frameworks to analyze trade in wildlife products.

### 2.1 Supply‐Side (Opportunity‐Based) Variables

#### 2.1.1 Concealability

Certain species may be more concealable than others, both at the market stage and at other stages such as the transport stage (Pires 2015; Moreto and Lemieux 2015). Body size may affect how difficult it is to hide an individual primate, whether in transport or within crates or stalls in open markets. Moreover, species that are legally trappable up to certain quotas (i.e., not fully protected under law within a country or region) may be more concealable along the trade chain and in markets, given that no one individual vendor is likely to hold the maximum number of individuals that can be legally trapped for one species (Gastañaga et al. 2011; Pires 2015). We can assume that species that are legally trappable in the focal country will likely be perceived as being of lower profile by enforcement, traders, and consumers than those that are given full protection under domestic laws, and thus may attract less attention (Kahler et al. 2022). In the open‐air markets of Indonesia, for example, vendors note that high‐profile protected species such as orangutans tend to draw more attention from law enforcement officers when compared to lower‐profile, or less protected, species (Shepherd 2010), although confiscations may remain low (Nijman 2017; Sherman et al. 2022).

#### 2.1.2 Availability: Abundance and Accessibility

Understanding the availability of a species in the wild is important in helping understand why the animal is hunted and why it may be over‐represented in a market (Moreto and Lemieux 2015; Pires 2015). Following research on the poaching and trade of other wildlife (e.g., parrots), availability of a primate species can be understood by two variables, *abundance* and *accessibility* (Pires and Clarke 2012). One indicator of abundance is a species' population trend, such as the decreasing, stable, or increasing population status as designated in the IUCN Red List of Threatened Species (IUCN 2025). It is assumed that species with higher abundance are often easier to encounter than species with lower abundance. Moreover, the IUCN threat category may serve as a proxy for how common the species is in the wild (IUCN 2025); species that are Critically Endangered are the least common and those of Least Concern may be deemed the most common.

Primates occur in seven different types of natural habitats: forest; savannah; shrubland; grassland; wetlands; rocky areas; and desert (Galán‐Acedo et al. 2019), as well as in anthropogenic habitats or human‐modified environments (e.g., urban systems, temples, agroforests, plantations) (IUCN 2025). While species may occur in more than one habitat, some habitats may be classified as more suitable or important than others for a species; this may be due to factors such as frequency of the species occurring in the habitat, number of individuals found in that habitat, or the requirement of the habitat at some point in the life cycle of the species (e.g., for breeding or foraging) (IUCN 2025). Some habitats may be more difficult for hunters and trappers to access than others (e.g., wetlands are more difficult to access compared to anthropogenic habitats; Nowak 2013; McLennan et al. 2017) or there may be increased access through encroachment from activities such as logging, mining, and agriculture (Remis and Jost Robinson 2012; McLennan et al. 2017), among other factors. Species that inhabit a catchment area closer to a market may also be more vulnerable to trapping and trade (Pires 2015). In sum, species that are both abundant and accessible are more susceptible to harvest and trade.

#### 2.1.3 Removability

Once a species has been located, its removability is important in understanding why that species may make its way from the source habitat to a market (Moreto and Lemieux 2015; Kahler et al. 2022). A primate's locomotion type refers to the species' main form of movement in its environment, including whether it is primarily arboreal, terrestrial, or commonly active on both the ground and in trees (Galán‐Acedo et al. 2019). Primarily arboreal primates may be more difficult to capture than terrestrial ones, but this may be affected by increased access to technologies such as guns (Remis and Jost Robinson 2012) or other aspects of primate behavioral ecology such as diel activity and social structures. The diel activity of a primate species can be categorized as diurnal (primarily active during the day), nocturnal (primarily active at night), or cathemeral (active both during day and nighttime) (Galán‐Acedo et al. 2019), and it can be assumed that nocturnal primates are more evasive to capture by humans than diurnal ones (Nekaris et al. 2020). Moreover, primate group sizes are highly variable and can range from one individual to more than 800 (IUCN 2025). Human hunting innovations such as use of technology (e.g., guns, cars, nets, snares) or dogs may make larger groups of primates more susceptible to hunting and increase the probability of capturing at least one individual during a trapping event (as discussed in Rivera et al. 2021).

### 2.2 Demand‐Side (Consumer‐Driven) Variables

#### 2.2.1 Processability

Some wildlife products may need to be altered or modified (processed) as they progress along a market chain, such as raw ivory altered to appear as wood or wild meat smoked or salted for concealment and preservation, with the final product that makes it to the market being different than that at the poaching stage (Moreto and Lemieux 2015; Kahler et al. 2022). Live primates may have some of their teeth removed so as to reduce the actual or perceived risk of harm to the consumer, as has been observed in the removal of the toothcomb and premolars in slow lorises and canines in siamangs and gibbons in Southeast Asian markets (Nijman et al. 2017). Teeth removal has also been observed in primates rescued from trade in the Peruvian Amazon (Rivera, personal observation).

#### 2.2.2 Replaceability (Transferability/Disposability)

A species that is consistently found across multiple markets within a focal area (regardless of the number of individuals of that species) may indicate higher demand by both consumers and market vendors (Pires 2015) and may also signify that the species is more easily replaceable. Moreto and Lemieux (2015) proposed that the demand‐side factor “transferability” replace “disposability” when examining products in markets, given that some products (e.g., ivory carvings) may be further traded, passed down, or given as gifts. In the case of live animals, we propose that the term “replaceability” adequately captures the demand‐side or consumer‐driven element of both the transferability and disposability of products in illicit trade. If individuals of a primate species are found in more than one market within a focal site (e.g., within a city) then the individuals of that species can be considered to be more easily replaced if given away, if they die due to poor conditions in captivity, or if they are disposed of in an attempt to hide the live animals from law enforcement. If the species is found in multiple markets, then individuals of that species can also be obtained from other vendors to replace stocks when there is an increase in demand.

#### 2.2.3 Usability

Primates are considered to be “difficult” or “extreme” to maintain in captivity due to their ecological needs and safety risks to humans (e.g., zoonotic disease transmission) (Warwick et al. 2014). However, primate species vary in their behavioral and ecological flexibility and ability to adapt to captive environments (McLennan et al. 2017; Stewart et al. 2025). For example, the success in captivity of a primate, or its tendency to have high or low mortality in captive environments, is linked to the ability of certain species to adapt to changes in their diet (Zehr et al. 2014). Thus, we assume that primates with higher ecological and behavioral flexibility may experience lower mortality rates in captivity and are thus more suited for different uses after purchased, including for co‐sharing space as a companion, pest control, entertainment, and income‐generating activities such as the photo prop trade (Norconk et al. 2020).

#### 2.2.4 Enjoyability

The enjoyability of a species to consumers can be measured by its attractiveness. For example, the enjoyability of traded parrots can be expressed by a composite measure that combines the body length, number of different plumage colors, the percentage of the body that is bright, and mimicry ability (Pires 2015; Pires and Petrossian 2016). Small primates are known to be prominent in the domestic pet trade due to their enjoyability as small companions that can be carried (Nijman et al. 2017; Norconk et al. 2020). Younger individuals, particularly those who have not reached sexual maturity, are also more desirable as pets given their perceived tamer behavior compared to that of adults (Soulsbury et al. 2009; Norconk et al. 2020). Moreover, data collected from pet‐trade websites and social media platforms (TikTok, YouTube) suggest that demand may be exacerbated through perceptions of and emphasis on the “cute,” “human‐like,” and “cuddly” characteristics of different primate species, among other terms used to advertise primates for sale (Moloney et al. 2021; Seaboch and Cahoon 2021; Collins and Campera 2024).

#### 2.2.5 Value (Desirability)

Diverse values can be attributed to primates based on the ecological, economic, and symbolic roles they play in human lives (Remis and Hardin 2009; Rivera et al. 2024). These include both monetary and nonmonetary values (Kahler et al. 2022). Ecological value may include additional value to humans beyond sharing space as a companion (pet), including pest control (e.g., keeping small primates to control insect pests in the home; Mere Roncal et al. 2018). Rarer species, including threatened and endemic species (i.e., species of conservation value), may hold higher economic and symbolic value and may be more vulnerable to trade than common and/or less threatened species (Alexander et al. 2023).

### 2.3 Framework Operationalization

Themes and variables in the proposed framework can be operationalized qualitatively as code groups and codes, respectively, for analyses of primate species vulnerability to hunting and trade in interview transcripts, ecological and ethnographic field notes, and literature reviews (Bernard et al. 2016) (Table 1 and Figure 1). As Blair, Le, Thạch, et al. (2017) note, the internal validity of qualitative data can complement the external validity of standardized quantitative data (e.g., market data). To operationalize the framework quantitatively, we provide a set of measurements for each of the variables to assess species vulnerability following a similar scoring system to analyze parrots observed in markets (Pires 2015; Pires and Petrossian 2016) (Table 2). The measurements allow for the scoring of each variable in the framework by species observed, and facilitate quantitative analyses to assess which variables may explain disparities in the numbers of observed individuals by species (i.e., why there are more individuals of certain species and fewer of others in urban markets). While some of the measurements in the quantitative framework (Table 2) are tailored to analyze the domestic trade of primates in Indonesia (given the example presented below), the variables and measurements are flexible enough to be adapted to other contexts.

**Table 2:** Framework to quantitatively examine primate species vulnerability to live trade, including independent variables to be measured by species.

Conservation criminology element	Variable	Measurement (score)	Assumptions
*Supply‐side (opportunity‐based)*			
Concealable	*Concealability* Composite measure that combines: *Legally trappable* + *Average body size*	*1–4 scale (1: least concealable; 4: most concealable)* 0 or 1 (1: legally trapped in country; concealable) + 1–3 scale (1: large body size; 2: medium body size; 3: small body size/most concealable)	Species that are legally trappable up to certain quotas (i.e., not fully protected) may be more concealable in markets. Smaller primates are more concealable. Body size can be scaled to small (0.03–5 kg), medium (5.1–10 kg), or large (> 10 kg) (Galán‐Acedo et al. 2019).
Available	*Abundance* Composite measure that combines: *Population trend* + *Threat category*	*2–8 scale (8: most abundant)* 1–3 scale (1: decreasing; 2: stable; 3: increasing) + 1–5 scale (1: Critically Endangered; 5: Least Concern)	Species with increasing population trends and least threatened (e.g., Least Concern on the IUCN Red List) are the most abundant.
	*Habitat access*	*1–10 scale (10: most accessible)* 1–10 scale [max of 10 if found in all four habitat categories] (1: wetland, most difficult habitat to access; 2: forest; 3: shrubland/grassland; 4: anthropogenic habitat, least difficult habitat to access)	Habitat access is scaled from most to least difficult habitat to access (Nowak 2013; McLennan et al. 2017). The scale is adjusted to include the types of primate habitat occurring in Indonesia (Galán‐Acedo et al. 2019; IUCN 2025). A species is scored as being found in a specific habitat if the habitat type is classified as being of “major importance” in the species' IUCN Red List profile (IUCN 2025). If the species is found in more than one habitat in the focal country, the scores are added.
	*Inhabitance in focal island*	*0 or 1 (1: found in focal island where market is located)*	Inhabitance in the focal island is used as a proxy for distance to market in Indonesia. Species found on the focal island are more accessible.
Removable	*Removability* Composite measure that combines: *Locomotion type* + *Diel activity* + *Group living*	*2–6 scale (6: most removable)* 1–3 scale (1: arboreal; 2: both; 3: terrestrial) + 1–2 scale (1: nocturnal; 2: diurnal) + 0 or 1 (0: solitary; 1: group living)	Arboreal primates are more difficult to capture, followed by primates that are both arboreal and terrestrial, and those that are primarily terrestrial. Nocturnal primates are more evasive to capture by humans than diurnal primates. Cathemeral activity is not documented among Indonesian primates (Galán‐Acedo et al. 2019). Primates living in groups may be more susceptible to capture and removal than those living solitarily.
*Demand‐side (consumer‐driven)*			
Processable	*Teeth removal*	*0 or 1 (1: most individuals observed in trade have their teeth removed)*	Some primates have their teeth removed so as to modify their perceived or actual threat to the consumer (Nijman et al. 2017).
Replaceable (transferable/disposable)	*No. of markets*	*0–N, where N is the max no. of markets where species is found (the highest N: most replaceable)*	A species that is found in multiple markets (regardless of individuals of that species) may be in more demand by both consumers and the market vendors (Pires 2015) and may be more easily replaced if the individual dies, is given away, or disposed of.
Useable	*Success in captivity* Composite measure that combines: *Trophic guild* + *Teeth removal*	*1–4 scale (4: more successful in captivity/low risk of mortality)* 1–3 scale (1: restricted diet (gummivore); 2: lower diet flexibility (frugivore; folivore; insectivore); 3: higher diet flexibility (omnivore; folivore–frugivore)) + 0 or 1 (0: teeth removed, high risk of mortality; 1: teeth not removed, low risk of mortality)	Primates with higher ecological flexibility (e.g., diet flexibility) and low mortality in captivity are more suited for different uses after purchase. The composite measure assumes that teeth removal leads to higher mortality. Primates which have their teeth removed are highly susceptible to infections, secondary abscesses, and premature death (Moore et al. 2014; Nijman et al. 2017).
	*Multiple uses*	*0 or 1 (1: more than one potential use after purchased live)*	Species with multiple uses after purchased live may have a higher demand (Kahler et al. 2022).
Enjoyable	*Enjoyability* Composite measure that combines: *Average body size* + *Age group traded*	*2–6 scale (6: most enjoyable)* 1–3 scale (1: large, 2: medium, 3: small) + 1–3 scale (1: mostly adults; 2: equal adults/nonadults; 3: mostly nonadults)	Smaller and younger primates may be perceived as more enjoyable (e.g., can be carried, are less aggressive).
Valuable/desirable	*Economic value*	*Average price*	The economic value of a product or species may signal a demand.
	*Ecological value*	*0 or 1 (1: holds additional ecological value to humans)*	Ecological value may include additional value to humans beyond sharing space as a companion (pet), including pest control or use of derivatives for protein or medicine (Kahler et al. 2022).
	*Conservation value* Composite measure that combines: *Threat status* + *Endemism*	*1–6 scale (6: highest conservation value/rarity)* 1–5 scale: (1: Least Concern; 5: Critically Endangered) + 0 or 1 (1: endemic to focal country)	Species that are Critically Endangered (IUCN 2025) and endemic to the focal country are rarer and of higher conservation value.
	*Symbolic/cultural value*	*0 or 1 (1: holds symbolic/cultural significance)*	Includes cultural roles and sociocultural values and meanings attributed to primates (Nekaris et al. 2010; Thạch et al. 2018; Rivera et al. 2024).

*Note:* Conservation criminology elements are derived from target‐suitability frameworks to analyze trade in wildlife products. The framework presented has been tailored to analyze live primates observed in domestic trade in Indonesia, but can be adapted to other contexts.

## 3 Example

### 3.1 Framework Adaptation and Data

We test the utility of our framework by applying it to a representative set of market data on live primate trade in the urban markets of Medan, North Sumatra, Indonesia. Indonesia is home to over 60 primate species, has been identified as a priority range country for primate conservation, and plays a major role in the international and domestic primate trade (Estrada et al. 2017, 2018; Alexander et al. 2023; Gamalo et al. 2024). Indonesian primates are some of the most imperiled on the planet with 83% of primate species threatened with extinction and about 94% of primate populations in decline (Estrada et al. 2017, 2018). The city of Medan in North Sumatra is considered a long‐standing hub for the primate and other wildlife trade (Shepherd et al. 2004; Nijman et al. 2017, 2021).

We first tailor some of the measurements in the quantitative framework to analyze domestic trade of primates in Indonesia (Table 2). For the variable *habitat access*, the types of habitats specified in the measurement can be modified to those found in the focal context and then scaled to difficulty of access. The variable *inhabitance in focal island* is proposed as a proxy for distance to market in Indonesia, assuming that species found within a focal island may be more likely to end up in markets on that island as opposed to markets in distant islands. Other studies have estimated distance to market using data on known sites of species harvest (Blair, Le, Thạch, et al. 2017), but these data are not always readily available. Cathemeral activity is not documented among Indonesian primates (Galán‐Acedo et al. 2019) and is thus not a factor considered for *diel activity* in the composite measure for *removability*. Similarly, monkeys of the Americas (Platyrrhines) are primarily arboreal, thus, it may not be necessary to account for variability in *locomotion type* when developing a numerical score for *removability* in market studies in the Americas. Lastly, we propose *teeth removal* as both a variable to measure *processability* and a factor in the composite measure for *success in captivity* (usability), given that some primates in Indonesian markets have their teeth removed to reduce risk to humans but are also left highly susceptible to infections, secondary abscesses, and premature death (Moore et al. 2014; Nijman et al. 2017). Studies on primate trade in other contexts should consider the extent to which live primates may be physically and/or behaviorally altered in different ways to meet consumer demand.

Shepherd (2010) conducted 66 surveys in three wildlife markets of Medan during the time period 1997–2008 and observed 1953 live primates of 10 species. Most of the individual primates detected in the surveys consisted of the following species: long‐tailed macaque (*Macaca fascicularis*, *n* = 774), greater slow loris (*Nycticebus coucang*, *n* = 714), pig‐tailed macaque (*Macaca nemestrina*, *n* = 380), and silvered leaf monkey (*Trachypithecus cristatus*, *n* = 65) (Shepherd 2010). There were fewer than 10 individuals of the following taxa detected: other leaf monkeys (*Trachypithecus auratus* [*T. auratus*], *Presbytis thomasi, Presbytis melalophos*), gibbons (*Hylobates agilis* [*H. agilis*], *Hylobates lar*), and siamang (a type of gibbon; *Symphalangus syndactylus*). While live quotas were allocated for the legal harvest and trade of four species at the country level (i.e., species were under regulated trade, e.g., to supply biomedical research), no quotas were allocated for trade in urban wildlife markets (i.e., physical markets where animals are openly or discreetly sold), thus the trade of all observed individuals in this study is considered illegal. The author reports on key and representative market variables that, when supplemented with published data on primate ecology and conservation, can be used to test the utility of our proposed framework, particularly within a specific temporal and geographic context. The variables of interest reported by Shepherd (2010) include: species observed; total number of observed individuals; national protected status; and IUCN Red List of Threatened Species status during the study period. For data on primate ecology and conservation, we referred to a database on the ecological traits of the world's primates (Galán‐Acedo et al. 2019) and species profiles on the IUCN Red List of Threatened Species (IUCN 2025). Studies analyzing historical and contemporary data using our framework should aim to use the IUCN Red List of Threatened Species Status and population trends of each species reported during the study period, if available.

We used the Google Scholar search engine to identify additional published literature on the primate trade in Sumatra so as to infer values for other parameters during the focal time period (e.g., age groups traded, teeth removal, symbolic value) and to contextualize the market data in time and space. We searched for articles and reports in English and Bahasa Indonesia.

### 3.2 Analysis

For each of the ten observed primate species, we calculated a score for each variable in the adapted CAPTURED framework (Tables 2 and 3; see Tables S1–S11 in the Supporting Material for score calculations). For any calculations that required IUCN Threat Status, we deferred to the status reported by Shepherd (2010) at the time of study. The resulting scores for each variable can be treated as independent variables for analysis while the dependent variable is the number of individual primates observed in markets classified by species (Table 3). We analyzed the relationships between the independent variables and the dependent variable to assess which factors explained the variability in primate species observed in the urban markets of Medan during the study period (1997–2008; Shepherd 2010). We used the R Statistical Software (https://www.r-project.org/) to conduct a principal component analysis (PCA; Agarwal et al. 2021) on the twelve variables for which we could calculate scores for the secondary market data (Table 3). We used PCA to account for potential correlations among multiple variables in our sample data set. Statistical significance was set at a *p* < 0.05. We contextualized the quantitative findings with the literature review on domestic primate trade in Sumatra.

**Table 3:** Measurements for conservation criminology variables to assess primate species vulnerability to illicit trade for observed species in urban markets in Medan, North Sumatra in 1997–2008 (Shepherd 2010).

				Supply‐side (opportunity‐based)					Demand‐side (consumer‐driven)						
Conservation criminology element					Available				Processable	Usable			Desirable/valuable		
Species (common name)	IUCN status (Shepherd 2010)	IUCN status (2025)	Market count	ConcealableConcealability (1–4)	Abundance (2–8)	Habitat access (1–10)	Focal island (0 or 1)	RemovableRemovability (2–6)	Teeth removal (0 or 1)	Success in captivity (1–4)	Multiple uses (0 or 1)	EnjoyableEnjoyability (2–6)	Ecological value (0 or 1)	Conservation value/rarity (1–6)	Symbolic value (0 or 1)
*Macaca fascicularis* (long‐tailed macaque)	LC	EN	774	4	5	7	1	5	0	3	1	6	1	1	0
*Nycticebus coucang* (greater slow loris)	VU	EN	714	3	3	6	1	2	1	3	1	5	1	3	0
*Macaca nemestrina* (pig‐tailed macaque)	VU	EN	380	3	3	6	1	5	0	3	1	5	1	3	0
*Trachypithecus cristatus* (silvered leaf monkey)	NT	VU	65	3	4	2	1	4	0	3	1	5	1	2	1
*Presbytis thomasi* (Thomas's leaf monkey)	VU	VU	7	2	3	2	1	4	0	3	0	5	0	4	1
*Presbytis melalophos* (Sumatran leaf monkey)	EN	EN	4	3	2	2	1	4	0	4	0	5	0	5	1
*Trachypithecus auratus* (ebony leaf monkey)	VU	VU	3	2	3	2	0	4	0	3	0	5	0	4	1
*Hylobates agilis* (agile gibbon)	EN	EN	3	2	2	2	1	4	1	2	0	5	0	4	1
*Symphalangus syndactylus* (siamang)	EN	EN	2	1	2	2	1	4	1	3	0	4	0	4	0
*Hylobates lar* (white‐handed gibbon)	EN	EN	1	2	2	2	1	4	1	2	0	5	0	4	1

*Note:* A total of 1953 individuals of ten primate species were detected during the study period. Values in parentheses indicate the measurement range for each variable. No data were available to measure the max no. of markets the species was traded in (replaceability/disposability) and average price (economic value), both demand‐side factors. IUCN Red List statuses reported by Shepherd (2010) during the focal time period were used for analyses.

IUCN Red List Threat Status Categories.

Abbreviations: EN, Endangered; LC, Least Concern; NT, Near Threatened; VU, Vulnerable.

### 3.3 Results and Discussion

Studies on the wildlife trade in Medan report that primates were sourced locally and trapped opportunistically, particularly in plantations and forests in the province of North Sumatra, with most species destined to supply the pet trade (Shepherd et al. 2004; Nekaris et al. 2010). Some species such as macaques and langurs were occasionally sold as food and some lorises were sold for medicinal use (Shepherd 2010). In some communities in North Sumatra, lorises are used in ritual practices. Burying a loris under a house or wearing its bones is believed to bring good luck and loris body parts are used to place curses on enemies (Nekaris et al. 2010), though there are no reports linking this type of sourcing to meet demand from urban residents. While no orangutans and only two siamangs were detected during market surveys in 1997–2008 (Shepherd 2010), vendors noted that they could be obtained and supplied upon request (Shepherd et al. 2004). The high prevalence of orangutans and gibbons in zoos and rescue centers in Sumatra pointed to a vibrant trade of these apes (Nijman 2009; Nijman et al. 2009).

Three agile gibbons (*H. agilis*), a species which is not native to North Sumatra, were detected in the focal market surveys (Shepherd 2010). The individuals were reportedly obtained from the province of Riau, highlighting interprovincial trade (Shepherd et al. 2004). In a sample of 34 lorises rescued from trade in Java, two‐thirds originated from Sumatra, also highlighting the sourcing to meet demand in other islands (Nekaris and Jaffe 2007). Three Javan silvered leaf monkey (or ebony leaf monkeys; *T. auratus*) were detected in the Medan market surveys and it is the only species known to have originated on another island (Shepherd et al. 2004). Overall, traded primates were often very young and lorises and gibbons had their teeth removed (Shepherd et al. 2004; Shepherd 2010).

**Figure 2:**
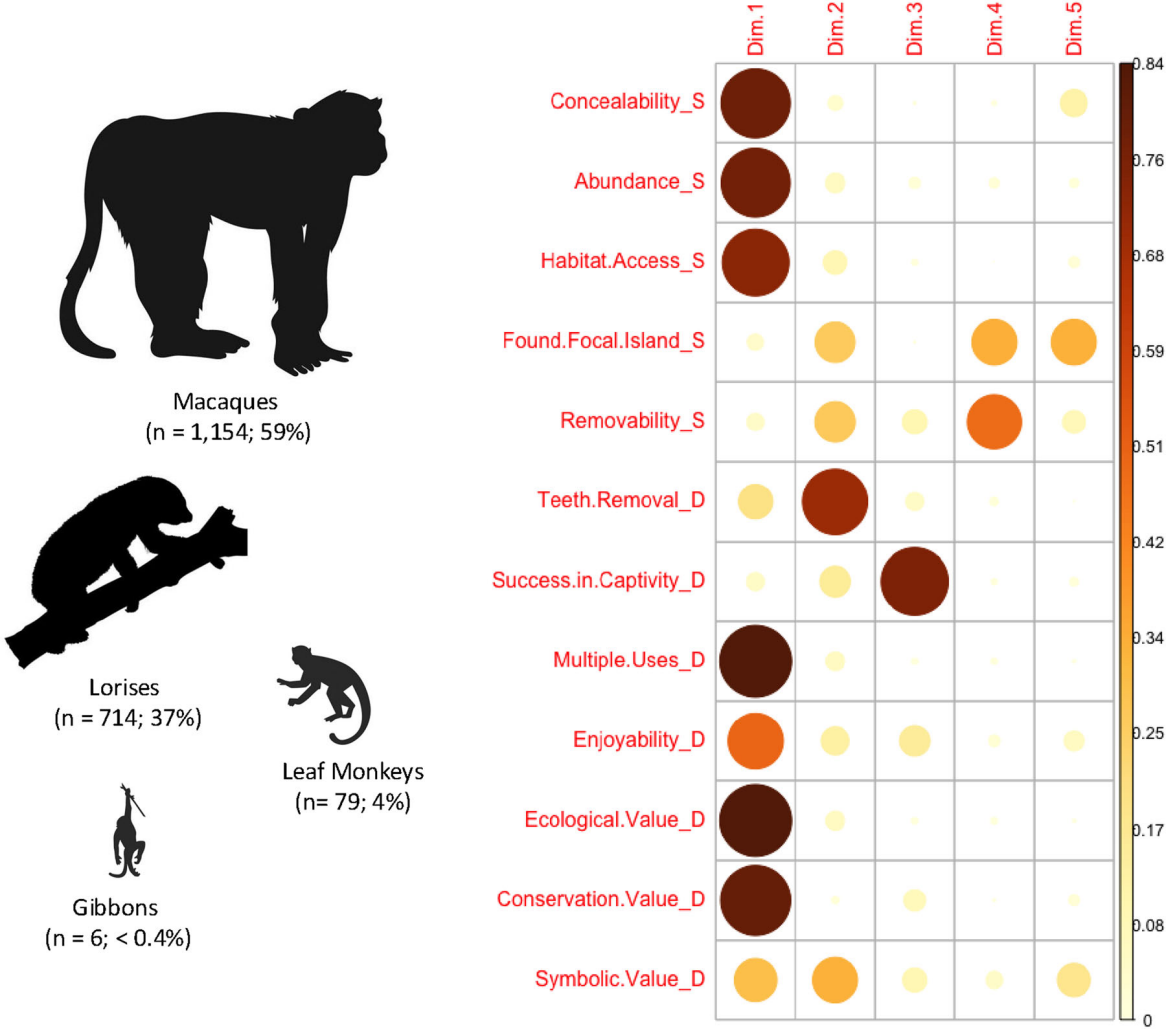
Variable correlation plot from a principal component regression to analyze the relationship between conservation criminology variables (to assess primate species vulnerability to illicit trade) and the number of individual primates detected in market surveys for ten species in Medan, North Sumatra, Indonesia (1997–2008; Shepherd 2010). D = demand‐side variable, S = supply‐side variable. The color scale (vertical color bar) and size of the circles represent the strength of association between the variables and principal components (larger and darker circles denote stronger associations).

In our quantitative analysis of species detected in Medan markets, the first principal component (PC1) significantly predicts most of the variance in the number of individual primates observed in the markets by species; PC1 accounts for 49.3% of the variability in the data (Figure S1). There is a positive relationship between PC1 and the dependent variable (number of individual primates in trade by species) (Table 4). The first principal component has large associations with three variables related to supply: *abundance* and *access to habitat* (both measures of “availability” in our framework) and *concealability* (composite measure of small body size and not being fully protected by law) (Figure 2 and Figure S1). PC1 also has large associations with the following demand‐side variables: *multiple uses* (more than one potential use after purchased live), *ecological value* (i.e., additional value beyond companionship, such as protein, medicine; related to *multiple uses*), and *conservation value or rarity* (Figure 2 and Figure S1). The association between PC1 and *conservation value or rarity* is negative (Figure S1). We can infer from our analysis that the aforementioned factors explained the vulnerability to trade and high detection of macaques and lorises in the wildlife markets of Medan when compared to the lower numbers of leaf monkey and gibbon species. The application of our framework also reveals that both demand‐ (opportunity‐based) and supply‐side (consumer‐driven) factors were drivers of the live primate trade in Medan during the focal time period (1997–2008) and social‐ecological context (Figure 2). No data were available to measure the replaceability (maximum number of markets each species was traded in) or economic value (average price) of each species, both demand‐side factors.

**Table 4:** Results of the principal component regression to analyze the relationship between conservation criminology variables to assess primate species vulnerability to illicit trade and number of individual primates detected in market surveys for ten species in Medan, North Sumatra (1997–2008; Shepherd 2010).

Variable	Coefficient	Std. error	*t*	Prob.
(Intercept)	195.30	43.87	4.45	0.00**
PC1	103.35	18.04	5.73	0.00***
PC2	−71.28	29.87	−2.39	0.05
*Residual standard error*	138.7 on 7 degrees of freedom			
*Multiple R^2^*	0.8462	*Adjusted R^2^*	0.80	
*F‐statistic*	19.26 on 2 and 7 DF	*p*	0.001	

*Note:* Significance level is set at *p* < 0.05.

Significance codes:

*** *p* < 0.001;

** *p* < 0.01.

Through this example, we elucidate the drivers of primate trade inferred from market data during 1997–2008, illustrating the utility of our framework in the analysis of previously compiled data sets. Even when parameters to measure certain variables are missing from those data sets, our approach offers a step toward further inquiry on the role of demand‐ and/or supply‐side drivers of trade within a context. Nevertheless, repeating market surveys at the same sites and analyzing data for the present temporal scale would allow for both comparisons in market changes over time (e.g., of observed species and drivers of trade) and the proposal of timely and context‐specific supply‐ and demand‐side interventions. Actualized surveys are particularly important given the continued prevalence of open wildlife trade in urban markets in Indonesia (Nijman et al. 2017; Nekaris and Nijman 2018; Alexander et al. 2023); revised taxonomies (e.g., slow lorises; Blair et al. 2023); updates in conservation status for certain species (e.g., long‐tailed macaques; Hansen et al. 2021; IUCN 2025); and potential impacts to trade dynamics from evolving human–primate relations and interactions (e.g., macaques considered a pest in North Sumatra; Kuswanda et al. 2023), among others. We also note that the results of the PCA should be interpreted with caution as a small sample size (of species, in this example) can produce limited principal components. Nevertheless, Indonesia is home to a high number of primate species, and it is likely that in other primate trade countries the detected number of species in urban markets may also be limited. Scaling to regional analyses (e.g., primate trade in Southeast Asian markets) may increase the interpretability of the results from a PCA.

The variables proposed in our framework can be used to guide and advance the design of market surveys, for example, through ensuring that both quantitative and qualitative data on the following site‐specific variables are collected: alteration/modification of the perceived or actual threat of individual primates to consumers; number of markets where each species is found; types of uses beyond companionship as a pet; and diverse values (economic, ecological, symbolic) attributed to species by urban vendors and consumers. The aforementioned market data, coupled with existing data on the ecological traits and conservation statuses of the world's primates (Galán‐Acedo et al. 2019; IUCN 2025), will ensure the operationalization of all variables in our framework and the contextualization of site‐specific market dynamics. From a conservation perspective, practitioners can use insights from analyses using the proposed framework to understand why human actors might make decisions to engage in sourcing, trading, or purchasing live primates of different species (Gore and Bennett 2022). Predictive assessments that reveal the multiple factors influencing a species' vulnerability to trade, such as those that result from operationalizing the CAPTURED framework, offer a more comprehensive understanding of the attributes of certain taxa that make them “hot products” within a given time and place. The identification of these attributes can help identify other vulnerable taxa that share similar attributes but which may not be visibly detected in open markets. Working toward disentangling these intertwined and taxon‐specific drivers will then inform whether investments in conservation actions to mitigate trade should focus on supply‐ and/or demand‐side interventions, such as mitigating human access to primate habitat or implementing behavior change campaigns to reduce primate pet ownership.

## 4 Comparison and Critique

Our framework is the first to illustrate the potential for integrating approaches in primatology and conservation criminology for analyses of primate species‐specific vulnerability to trade. Studies of mammal vulnerability to hunting and use by humans have traditionally focused on biological traits such as body mass (e.g., Benítez‐López et al. 2019). Our framework is primate‐focused and explicitly incorporates biological and social considerations within a vulnerability framework that discriminates between supply‐ and demand‐side variables. This contributes to calls for research that integrates social and ecological variables in studies of drivers of hunting and trade of primates (e.g., Blair, Le, Thạch, et al. 2017; Rivera et al. 2021). The qualitative and quantitative adaptations of our framework are flexible and can be adapted to study both legal and illegal trade of primates across diverse market contexts. Our list of supply‐ (opportunity‐based) and demand‐side (consumer‐driven) variables serves as a list of considerations in market survey design and as a starting point for identification of additional context‐relevant variables to measure. Continued innovation in the integration of ecological and conservation science with crime sciences will offer paths toward sustainable management of human–primate interactions and reductions in wildlife crime (Gore and Bennett 2022).

In some contexts, there is continued trade despite the low visibility of live primates sold in open markets. For example, in the urbanizing Peruvian Amazon, fewer primates are visible in open market stalls compared to the year prior to the COVID‐19 pandemic, yet vendors are willing to supply any taxa requested by urban residents and tourists (D'Cruze et al. 2021; Rivera et al. 2024; Rivera, unpublished data). Moreover, there is a growing trade in primates on online platforms; these online markets are low to no cost to access and can reach a broad range of potential consumers (Norconk et al. 2020; Nijman et al. 2021; Alexander et al. 2023). In Indonesia, the growing online primate trade is similar to that of the open market trade in that they both aim to meet domestic demand for live individuals (Nijman et al. 2021). Alexander et al. (2023) assert that despite growing cases of shifts of the trade to online platforms, urban contexts and their residents are still important in driving demand due to the ability of wealthier consumers to purchase live animals at a range of prices and reliable access to the internet (when compared to rural areas). Our framework is flexible enough to examine primates detected in online market platforms, particularly because sale advertisements and viewer captions may reveal market and taxa‐specific information related to supply‐ and demand‐side factors (e.g., the potential enjoyability of keeping lorises as pets as depicted in YouTube videos; Nekaris et al. 2013; Nijman et al. 2021; Freund et al. 2024).

While our framework is designed to be adaptable, we identify four variables that are particularly context‐specific: processability, multiple uses, ecological value, and symbolic or cultural value. In our empirical case study in Medan, Indonesia, these variables are defined according to local sociocultural practices, which in turn may limit the generalizability of the Indonesia‐focused framework when applied to other regions. Future applications of this framework should therefore redefine and adapt these variables to reflect socioeconomic and cultural contexts. Moreover, our scale for body size (small, medium, large; Table 2), which is partly used to calculate concealability, is based on categories defined by Galán‐Acedo et al. (2019). These categories are appropriate to use in the context of Indonesia where there is potential to detect primates as small as tarsiers and as big as orangutans in trade. Future researchers can adapt the scales for other contexts (physical or online) in which the range of body sizes expected to be observed may differ.

Both market data and primate ecological trait data (Galán‐Acedo et al. 2019) are conducive to relatively simple analysis using our framework and thus rapid assessments of drivers of wildlife trade. However, solely applying the quantitative adaptation of our framework to these data may limit our understanding of the complexity of primate species vulnerability across diverse primate trade systems (Blair, Le, Thạch, et al. 2017). For example, primate symbolic values and meanings are not static and can transform across social‐ecological landscapes (Peterson and Riley 2012). We advocate for concurrent ethnographic and ethnoprimatological studies when designing and carrying out market surveys toward identifying complex and underlying drivers of trade that cannot be elucidated through a solely quantitative assessment (Nekaris et al. 2010; Dore et al. 2018; Thạch et al. 2018). Such an approach would offer a more complete understanding of why more individuals of certain species are observed in markets over others and would guide the allocation of resources toward supply‐ and/or demand‐side interventions to mitigate multiple drivers of trade across diverse hunting and trade systems.

## Ethics Statement

This research presents a conceptual framework with analysis of secondary data, and as such did not require approval by the authors from an Institutional Animal Care and Use Committee or Institutional Review Board. The research adhered to the American Society of Primatologists (ASP) Principles for the Ethical Treatment of Non‐Human Primates.

## Supporting information


**Figure S1:** Variable correlation circle from Principal Component Analysis. **Tables S1–S11:** Calculations for conservation criminology variable scores of species observed in urban markets in Medan, North Sumatra, in 1997–2008 (Shepherd 2010).

## References

[ajp70102-bib-0001] Agarwal, A. , D. Shah , D. Shen , and D. Song . 2021. “On Robustness of Principal Component Regression.” Journal of the American Statistical Association 116, no. 536: 1731–1745. 10.1080/01621459.2021.1928513.

[ajp70102-bib-0002] Alexander, S. D. , S. Waters , B. C. Aldrich , et al. 2023. “The Past, Present, and Future of the Primate Pet Trade.” In Primates in Anthropogenic Landscapes: Exploring Primate Behavioural Flexibility Across Human Contexts, 247–266. Springer International Publishing.

[ajp70102-bib-0003] Badihi, G. , D. R. K. Nielsen , P. A. Garber , et al. 2024. “Perspectives on Conservation Impacts of the Global Primate Trade.” International Journal of Primatology 45, no. 4: 972–999.

[ajp70102-bib-0004] Benítez‐López, A. , L. Santini , A. M. Schipper , M. Busana , and M. A. J. Huijbregts . 2019. “Intact but Empty Forests? Patterns of Hunting‐Induced Mammal Defaunation in the Tropics.” PLoS Biology 17, no. 5: e3000247. 31086365 10.1371/journal.pbio.3000247 PMC6516652

[ajp70102-bib-0005] Bernard, H. R. , A. Wutich , and G. W. Ryan . 2016. Analyzing Qualitative Data: Systematic Approaches. SAGE Publications.

[ajp70102-bib-0006] Blair, M. , G. Cao , E. López‐Nandam , et al. 2023. “Molecular Phylogenetic Relationships and Unveiling Novel Genetic Diversity Among Slow and Pygmy Lorises, Including Resurrection of *Xanthonycticebus intermedius* .” Genes 14, no. 3: 643. 36980915 10.3390/genes14030643 PMC10048081

[ajp70102-bib-0007] Blair, M. E. , M. D. Le , and E. J. Sterling . 2017. “Multidisciplinary Studies of Wildlife Trade in Primates: Challenges and Priorities.” American Journal of Primatology 79, no. 11: e22710. 10.1002/ajp.22710 29023874

[ajp70102-bib-0008] Blair, M. E. , M. D. Le , H. M. Thạch , et al. 2017. “Applying Systems Thinking to Inform Studies of Wildlife Trade in Primates.” American Journal of Primatology 79, no. 11: e22715. 10.1002/ajp.22715 29035006

[ajp70102-bib-0009] Collins, D. , and M. Campera . 2024. “Investigating the Extent and Nature of the Primate Pet Trade on TikTok.” Conservation 4, no. 4: 547–559.

[ajp70102-bib-0010] D'Cruze, N. , F. E. R. Galarza , O. Broche , et al. 2021. “Characterizing Trade at the Largest Wildlife Market of Amazonian Peru.” Global Ecology and Conservation 28: e01631.

[ajp70102-bib-0011] Dore, K. M. , L. Radford , S. Alexander , and S. Waters . 2018. “Ethnographic Approaches in Primatology.” Folia Primatologica 89, no. 1: 5–12. 10.1159/000485693 29631259

[ajp70102-bib-0012] Estrada, A. , P. A. Garber , R. A. Mittermeier , et al. 2018. “Primates in Peril: The Significance of Brazil, Madagascar, Indonesia and the Democratic Republic of the Congo for Global Primate Conservation.” PeerJ 6: e4869. 29922508 10.7717/peerj.4869 PMC6005167

[ajp70102-bib-0013] Estrada, A. , P. A. Garber , A. B. Rylands , et al. 2017. “Impending Extinction Crisis of the World's Primates: Why Primates Matter.” Science Advances 3, no. 1: e1600946. 28116351 10.1126/sciadv.1600946 PMC5242557

[ajp70102-bib-0014] Freund, C. A. , K. A. Cronin , M. Huang , N. J. Robinson , B. Yoo , and A. L. DiGiorgio . 2024. “Effects of Captions on Viewers' Perceptions of Images Depicting Human−Primate Interaction.” Conservation Biology 38, no. 3: e14199. 37811716 10.1111/cobi.14199

[ajp70102-bib-0015] Galán‐Acedo, C. , V. Arroyo‐Rodríguez , E. Andresen , and R. Arasa‐Gisbert . 2019. “Ecological Traits of the World's Primates.” Scientific Data 6, no. 1: 55. 31086194 10.1038/s41597-019-0059-9 PMC6513815

[ajp70102-bib-0016] Gamalo, L. E. , K. Ilham , L. Jones‐Engel , et al. 2024. “Removal From the Wild Endangers the Once Widespread Long‐Tailed Macaque.” American Journal of Primatology 86, no. 3: e23547. 37667504 10.1002/ajp.23547

[ajp70102-bib-0017] Garber, P. A. , A. Estrada , S. Shanee , et al. 2024. “Global Wildlife Trade and Trafficking Contribute to the World's Nonhuman Primate Conservation Crisis.” Frontiers in Conservation Science 5: 1400613.

[ajp70102-bib-0018] Gastañaga, M. , R. MacLeod , B. Hennessey , et al. 2011. “A Study of the Parrot Trade in Peru and the Potential Importance of Internal Trade for Threatened Species.” Bird Conservation International 21, no. 1: 76–85.

[ajp70102-bib-0019] Gibbs, C. , M. L. Gore , E. F. McGarrell , and L. Rivers . 2010. “Introducing Conservation Criminology: Towards Interdisciplinary Scholarship on Environmental Crimes and Risks.” British Journal of Criminology 50, no. 1: 124–144.

[ajp70102-bib-0020] Gore, M. L. , and A. Bennett . 2022. “Importance of Deepening Integration of Crime and Conservation Sciences.” Conservation Biology 36, no. 1: e13710. 33600003 10.1111/cobi.13710 PMC9291754

[ajp70102-bib-0021] Hansen, M. F. , M. Gill , V. A. Nawangsari , et al. 2021. “Conservation of Long‐Tailed Macaques: Implications of the Updated IUCN Status and the COVID‐19 Pandemic.” Primate Conservation 35: 1–11.

[ajp70102-bib-0022] IUCN . 2025. *IUCN Red List of Threatened Species*. Version 2021‐3. International Union for Conservation of Nature. www.iucnredlist.org.

[ajp70102-bib-0023] Kahler, J. S. , C. J. Rivera , and M. L. Gore . 2022. “Introducing IPOACHED: A Conservation Criminology‐Based Framework to Understand Wildlife Species Targeted by Poachers in Protected Areas.” Frontiers in Conservation Science 3: 992621.

[ajp70102-bib-0024] Kahler, J. S. , J. W. Rivera , Z. T. Steele , et al. 2021. “Advancing Applied Research in Conservation Criminology Through the Evaluation of Corruption Prevention, Enhancing Compliance, and Reducing Recidivism.” Frontiers in Conservation Science 2: 698755.

[ajp70102-bib-0025] Kuswanda, W. , F. J. Hutapea , and T. Setyawati . 2023. “The Endangered Long‐Tailed Macaque Is Considered a Pest in North Sumatra, Indonesia.” Oryx 57, no. 1: 12–13.

[ajp70102-bib-0026] Linder, J. M. , S. C. Sawyer , and J. S. Brashares . 2013. “Primates in Trade.” In Primate Ecology and Conservation: A Handbook of Techniques, edited by E. Sterling , N. Bynum , and M. Blair , 323–345. Oxford. 10.1093/acprof:oso/9780199659449.003.0018.

[ajp70102-bib-0027] McLennan, M. R. , N. Spagnoletti , and K. J. Hockings . 2017. “The Implications of Primate Behavioral Flexibility for Sustainable Human–Primate Coexistence in Anthropogenic Habitats.” International Journal of Primatology 38: 105–121.

[ajp70102-bib-0028] Mere Roncal, C. , M. Bowler , and M. P. Gilmore . 2018. “The Ethnoprimatology of the Maijuna of the Peruvian Amazon and Implications for Primate Conservation.” Journal of Ethnobiology and Ethnomedicine 14: 19. 29514692 10.1186/s13002-018-0207-x PMC5842639

[ajp70102-bib-0029] Moloney, G. K. , J. Tuke , E. Dal Grande , T. Nielsen , and A. L. Chaber . 2021. “Is YouTube Promoting the Exotic Pet Trade? Analysis of the Global Public Perception of Popular Youtube Videos Featuring Threatened Exotic Animals.” PLoS One 16, no. 4: e0235451. 33848287 10.1371/journal.pone.0235451 PMC8043400

[ajp70102-bib-0030] Moore, R. , K. A. I. Wihermanto , and K. Nekaris . 2014. “Compassionate Conservation, Rehabilitation and Translocation of Indonesian Slow Lorises.” Endangered Species Research 26, no. 2: 93–102.

[ajp70102-bib-0031] Moreto, W. D. , R. W. Charlton , S. E. DeWitt , and C. M. Burton . 2020. “The Convergence of CAPTURED Fish and People: Examining the Symbiotic Nature of Labor Trafficking and Illegal, Unreported and Unregulated Fishing.” Deviant Behavior 41, no. 6: 733–749.

[ajp70102-bib-0032] Moreto, W. D. , and A. M. Lemieux . 2015. “From CRAVED to CAPTURED: Introducing a Product‐Based Framework to Examine Illegal Wildlife Markets.” European Journal on Criminal Policy and Research 21: 303–320.

[ajp70102-bib-0033] Nekaris, K. A. I. , N. Campbell , T. G. Coggins , E. J. Rode , and V. Nijman . 2013. “Tickled to Death: Analysing Public Perceptions of ‘Cute’ Videos of Threatened Species (Slow Lorises–*Nycticebus* spp.) on Web 2.0 Sites.” PLoS One 8, no. 7: e69215. 23894432 10.1371/journal.pone.0069215 PMC3722300

[ajp70102-bib-0034] Nekaris, K. A. I. , and S. Jaffe . 2007. “Unexpected Diversity of Slow Lorises (*Nycticebus* spp.) Within the Javan Pet Trade: Implications for Slow Loris Taxonomy.” Contributions to Zoology 76, no. 3: 187–196.

[ajp70102-bib-0035] Nekaris, K. A. I. , R. A. Munds , and E. Pimley . 2020. “Trapping, Collaring and Monitoring the Lorisinae of Asia (*Loris, Nycticebus*) and Perodicticinae (Arctocebus, Perodicticus) of Africa.” In Evolution, Ecology and Conservation of Lorises and Pottos: 279–294. Cambridge Studies in Biological and Evolutionary Anthropology.

[ajp70102-bib-0036] Nekaris, K. A. I. , and V. Nijman . 2018. “Successful Prosecution of Slow Loris Traders in Indonesia.” Oryx 52, no. 3: 411.

[ajp70102-bib-0037] Nekaris, K. A. I. , C. R. Shepherd , C. R. Starr , and V. Nijman . 2010. “Exploring Cultural Drivers for Wildlife Trade via an Ethnoprimatological Approach: A Case Study of Slender and Slow Lorises (*Loris* and *Nycticebus*) in South and Southeast Asia.” American Journal of Primatology 72, no. 10: 877–886. 20806336 10.1002/ajp.20842

[ajp70102-bib-0038] Nijman, V. 2009. An Assessment of Trade in Gibbons and Orang‐Utans in Sumatra, Indonesia, vii. TRAFFIC Southeast Asia.

[ajp70102-bib-0039] Nijman, V. 2017. “Orangutan Trade, Confiscations, and Lack of Prosecutions in Indonesia.” American Journal of Primatology 79, no. 11: 22652. 10.1002/ajp.22652 28407279

[ajp70102-bib-0040] Nijman, V. , C. F. Y. Martinez , and C. Shepherd . 2009. “Saved From Trade: Donated and Confiscated Gibbons in Zoos and Rescue Centres in Indonesia.” Endangered Species Research 9, no. 2: 151–157.

[ajp70102-bib-0041] Nijman, V. , T. Q. Morcatty , H. R. El Bizri , et al. 2023. “Global Online Trade in Primates for Pets.” Environmental Development 48: 100925.

[ajp70102-bib-0042] Nijman, V. , K. Nekaris , G. Donati , M. Bruford , and J. Fa . 2011. “Primate Conservation: Measuring and Mitigating Trade in Primates.” Endangered Species Research 13, no. 2: 159–161.

[ajp70102-bib-0043] Nijman, V. , J. H. Smith , G. Foreman , M. Campera , K. Feddema , and K. A. I. Nekaris . 2021. “Monitoring the Trade of Legally Protected Wildlife on Facebook and Instagram Illustrated by the Advertising and Sale of Apes in Indonesia.” Diversity 13, no. 6: 236.

[ajp70102-bib-0044] Nijman, V. , D. Spaan , E. J. Rode‐Margono , I. Wirdateti , and K. A. I. Nekaris . 2017. “Changes in the Primate Trade in Indonesian Wildlife Markets Over a 25‐Year Period: Fewer Apes and Langurs, More Macaques, and Slow Lorises.” American Journal of Primatology 79, no. 11: e22517. 10.1002/ajp.22517 26713673

[ajp70102-bib-0045] Norconk, M. A. , S. Atsalis , G. Tully , et al. 2020. “Reducing the Primate Pet Trade: Actions for Primatologists.” American Journal of Primatology 82, no. 1: e23079. 31876316 10.1002/ajp.23079 PMC9286354

[ajp70102-bib-0046] Nowak, K. 2013. “Mangrove and Peat Swamp Forests: Refuge Habitats for Primates and Felids.” Folia Primatologica 83, no. 3–6: 361–376. 10.1159/000339810 23363595

[ajp70102-bib-0047] Peterson, J. V. , and E. P. Riley . 2012. “ *Monyet Yang Dihargai, Monyet Yang Dibenci*: The Human‐Macaque Interface in Indonesia.” In The Macaque Connection: Cooperation and Conflict Between Humans and Macaques, 149–166. Springer New York.

[ajp70102-bib-0048] Petrossian, G. A. , and R. V. Clarke . 2014. “Explaining and Controlling Illegal Commercial Fishing: An Application of the CRAVED Theft Model.” British Journal of Criminology 54, no. 1: 73–90.

[ajp70102-bib-0049] Pires, S. F. 2015. “A CRAVED Analysis of Multiple Illicit Parrot Markets in Peru and Bolivia.” European Journal on Criminal Policy and Research 21: 321–336.

[ajp70102-bib-0050] Pires, S. , and R. V. Clarke . 2012. “Are Parrots CRAVED? An Analysis of Parrot Poaching in Mexico.” Journal of Research in Crime and Delinquency 49, no. 1: 122–146.

[ajp70102-bib-0051] Pires, S. F. , and G. A. Petrossian . 2016. “Understanding Parrot Trafficking Between Illicit Markets in Bolivia: An Application of the CRAVED Model.” International Journal of Comparative and Applied Criminal Justice 40, no. 1: 63–77.

[ajp70102-bib-0052] Pires, S. F. , G. Olah , D. Nandika , D. Agustina , and R. Heinsohn . 2021. “What Drives the Illegal Parrot Trade? Applying a Criminological Model to Market and Seizure Data in Indonesia.” Biological Conservation 257: 109098.

[ajp70102-bib-0053] Remis, M. J. , and R. Hardin . 2009. “Transvalued Species in an African Forest.” Conservation Biology 23, no. 6: 1588–1596. 19604297 10.1111/j.1523-1739.2009.01290.x

[ajp70102-bib-0054] Remis, M. J. , and C. A. Jost Robinson . 2012. “Reductions in Primate Abundance and Diversity in a Multiuse Protected Area: Synergistic Impacts of Hunting and Logging in a Congo Basin Forest.” American Journal of Primatology 74, no. 7: 602–612. 22644576 10.1002/ajp.22012

[ajp70102-bib-0055] Rivera, C. J. , A. Fuentes , and V. Hull . 2024. “Cultural Roles of Primates in an Amazonian Urban Center.” Journal for Nature Conservation 78: 126548.

[ajp70102-bib-0056] Rivera, C. J. , D. Mayo , and V. Hull . 2021. “Social‐Ecological Interactions Influencing Primate Harvest: Insights From Madagascar.” Frontiers in Conservation Science 2: 776897.

[ajp70102-bib-0057] Seaboch, M. S. , and S. N. Cahoon . 2021. “Pet Primates for Sale in the United States.” PLoS One 16, no. 9: e0256552. 34496001 10.1371/journal.pone.0256552 PMC8425555

[ajp70102-bib-0058] Shepherd, C. R. 2010. “Illegal Primate Trade in Indonesia Exemplified by Surveys Carried Out Over a Decade in North Sumatra.” Endangered Species Research 11, no. 3: 201–205.

[ajp70102-bib-0059] Shepherd, C. R. , J. Sukumaran , and S. A. Wich . 2004. Open Season: An Analysis of the Pet Trade in Medan, Sumatra 1997‐2001. TRAFFIC Southeast Asia.

[ajp70102-bib-0060] Sherman, J. , M. Voigt , M. Ancrenaz , et al. 2022. “Orangutan Killing and Trade in Indonesia: Wildlife Crime, Enforcement, and Deterrence Patterns.” Biological Conservation 276: 109744.

[ajp70102-bib-0061] Soulsbury, C. D. , G. Iossa , S. Kennell , and S. Harris . 2009. “The Welfare and Suitability of Primates Kept as Pets.” Journal of Applied Animal Welfare Science 12, no. 1: 1–20. 19107661 10.1080/10888700802536483

[ajp70102-bib-0062] Stewart, B. M. , M. M. Joyce , J. Creeggan , S. Eccles , M. G. Gerwing , and S. E. Turner . 2025. “Primates and Disability: Behavioral Flexibility and Implications for Resilience to Environmental Change.” American Journal of Primatology 87, no. 1: e23579. 38050800 10.1002/ajp.23579 PMC11650948

[ajp70102-bib-0063] Thạch, H. M. , M. D. Le , N. B. Vũ , et al. 2018. “Slow Loris Trade in Vietnam: Exploring Diverse Knowledge and Values.” Folia Primatologica 89, no. 1: 45–62. 10.1159/000481196 29631261

[ajp70102-bib-0064] Warwick, C. , C. Steedman , M. Jessop , E. Toland , and S. Lindley . 2014. “Assigning Degrees of Ease or Difficulty for Pet Animal Maintenance: The EMODE System Concept.” Journal of Agricultural and Environmental Ethics 27, no. 1: 87–101.

[ajp70102-bib-0065] Wilson, L. , and J. Kurland . 2019. “An Agenda for Criminological Investigation of Crimes Impacting Primates.” In Quantitative Studies in Green and Conservation Criminology, 71–86. Routledge.

[ajp70102-bib-0066] Zehr, S. M. , R. G. Roach , D. Haring , J. Taylor , F. H. Cameron , and A. D. Yoder . 2014. “Life History Profiles for 27 Strepsirrhine Primate Taxa Generated Using Captive Data From the Duke Lemur Center.” Scientific Data 1, no. 1: 140019. 25977776 10.1038/sdata.2014.19 PMC4322587

