# Supplementary material for: A Framework to Analyze Primate Species Vulnerability to Trade in Urban Markets
Source: Am J Primatol. 2025 Dec 7;87(12):e70102. doi: 10.1002/ajp.70102 (PMC12682214; doi:10.1002/ajp.70102)
Supplement: Supplementary file 1 — Figure S1: Variable correlation circle from Principal Component Analysis. Tables S1–S11: Calculations for conservation criminology variable scores of species observed in urban markets in Medan, North Sumatra, in 1997–2008 (Shepherd 2010). [file AJP-87-e70102-s001.pdf]

Supporting Information

TITLE: A framework to analyze primate species vulnerability to trade in urban markets

AUTHORS: Christian J. Rivera [1 2 3]; Jessica S. Kahler [4]; Wahyu Nurbandi [5]; Agustín Fuentes [3]

AFFILIATIONS:

[1] School for the Environment, University of Massachusetts Boston, Boston, MA, USA
[2] High Meadows Environmental Institute, Princeton University, Princeton, NJ, USA
[3] Department of Anthropology, Princeton University, Princeton, NJ, USA
[4] Department of Sociology and Criminology & Law, University of Florida, Gainesville, FL, USA
[5] Princeton School of Public and International Affairs, Princeton University, Princeton, NJ, USA

Corresponding Author: Christian J. Rivera
Address: School for the Environment, 100 Morrissey Blvd., Boston, MA 02125
Email: cj.rivera@umb.edu

Table S1. Calculations for **CONCEALABILITY** scores of species observed in urban markets in Medan, North Sumatra, in 1997-2008 (Shepherd, 2010).

**Species** (Shepherd, 2010)	**Common Name** (Shepherd, 2010)	**Legally trappable?** (Shepherd, 2010)	**LEGALLY TRAPPABLE SCORE** 1: legally trappable (not fully protected)	**Avg. Body Mass (kg)** (Galan-Acedo et al. 2019)	**AVG. BODY MASS SCORE** 1: large; 2: medium; 3: small	**CONCEALABILITY SCORE**
Nycticebus coucang	Greater slow loris	No	0	0.65	3	**3**
Macaca fascicularis	Long-tailed macaque	Yes	1	4.03	3	**4**
Macaca nemestrina	Pig-tailed macaque	Yes	1	8.89	2	**3**
Trachypithecus cristatus	Silvered leaf monkey	Yes	1	6.25	2	**3**
Trachypithecus auratus	Ebony leaf monkey	No	0	7.1	2	**2**
Presbytis thomasi	Thomas's leaf monkey	No	0	6.73	2	**2**
Presbytis melalophos	Sumatran leaf monkey	Yes	1	6.53	2	**3**
Symphalangus syndactylus	Siamang	No	0	11.3	1	**1**
Hylobates agilis	Agile gibbon	No	0	6	2	**2**
Hylobates lar	White-handed gibbon	No	0	5.64	2	**2**

Table S2. Calculations for **Availability: ABUNDANCE** scores of species observed in urban markets in Medan, North Sumatra, in 1997-2008 (Shepherd, 2010). We used historical IUCN Threat Statuses reported at the time of data collection (Shepherd, 2010). No data were reported for Population Trend of each species during the study period. Thus, we omitted the population trend score from the calculation of the abundance score; in this example, IUCN Status then serves a proxy for abundance.

**Species** (Shepherd, 2010)	**Common Name** (Shepherd, 2010)	**POPULATION TREND SCORE** 1: decreasing; 2: stable; 3: increasing	**IUCN Threat Status** (Shepherd, 2010)	**THREAT STATUS SCORE** 1: CR; 2: EN; 3: VU; 4: NT; 5: LC	**ABUNDANCE SCORE**
Nycticebus coucang	Greater slow loris	NA	Vulnerable (VU)	3	**3**
Macaca fascicularis	Long-tailed macaque	NA	Least Concern (LC)	5	**5**
Macaca nemestrina	Pig-tailed macaque	NA	Vulnerable (VU)	3	**3**
Trachypithecus cristatus	Silvered leaf monkey	NA	Near Threatened (NT)	4	**4**
Trachypithecus auratus	Ebony leaf monkey	NA	Vulnerable (VU)	3	**3**
Presbytis thomasi	Thomas's leaf monkey	NA	Vulnerable (VU)	3	**3**
Presbytis melalophos	Sumatran leaf monkey	NA	Endangered (EN)	2	**2**
Symphalangus syndactylus	Siamang	NA	Endangered (EN)	2	**2**
Hylobates agilis	Agile gibbon	NA	Endangered (EN)	2	**2**
Hylobates lar	White-handed gibbon	NA	Endangered (EN)	2	**2**

Table S3. Calculations for **Availability: HABITAT ACCESS** and **INHABITANCE IN FOCAL ISLAND** scores of species observed in urban markets in Medan, North Sumatra, in 1997-2008 (Shepherd, 2010).

		**HABITAT SCORE (Galan-Acedo et al. 2019; IUCN, 2025)** 0: not found in habitat/ not habitat of major importance						
**Species** (Shepherd, 2010)	**Common Name** (Shepherd, 2010)	**Wetland (1)**	**Forest (2)**	**Shrubland/ grassland (3)**	**Anthropogenic habitat (4)**	**HABITAT ACCESS SCORE**	**Inhabits focal island (Sumatra)?** (IUCN, 2025)	**FOCAL ISLAND SCORE**
Nycticebus coucang	Greater slow loris	0	2	0	4	**6**	Yes	**1**
Macaca fascicularis	Long-tailed macaque	1	2	0	4	**7**	Yes	**1**
Macaca nemestrina	Pig-tailed macaque	0	2	0	4	**6**	Yes	**1**
Trachypithecus cristatus	Silvered leaf monkey	0	2	0	0	**2**	Yes	**1**
Trachypithecus auratus	Ebony leaf monkey	0	2	0	0	**2**	No	**0**
Presbytis thomasi	Thomas's leaf monkey	0	2	0	0	**2**	Yes	**1**
Presbytis melalophos	Sumatran leaf monkey	0	2	0	0	**2**	Yes	**1**
Symphalangus syndactylus	Siamang	0	2	0	0	**2**	Yes	**1**
Hylobates agilis	Agile gibbon	0	2	0	0	**2**	Yes	**1**
Hylobates lar	White-handed gibbon	0	2	0	0	**2**	Yes	**1**

Table S4. Calculations for **REMOVABILITY** scores of species observed in urban markets in Medan, North Sumatra, in 1997-2008 (Shepherd, 2010).

**Species** (Shepherd, 2010)	**Common Name** (Shepherd, 2010)	**Locomotion Type** (Galan-Acedo et al. 2019)	**LOCOMOTION SCORE** 1: arboreal; 2: both; 3: terrestrial	**Diel Activity** (Galan-Acedo et al. 2019)	**DIEL ACTIVITY SCORE**	**Lives in groups?** (IUCN, 2025)	**GROUP LIVING SCORE** 1: yes	**REMOVABILITY SCORE**
Nycticebus coucang	Greater slow loris	Arboreal	1	Nocturnal	1	No	0	**2**
Macaca fascicularis	Long-tailed macaque	Both	2	Diurnal	2	Yes	1	**5**
Macaca nemestrina	Pig-tailed macaque	Both	2	Diurnal	2	Yes	1	**5**
Trachypithecus cristatus	Silvered leaf monkey	Arboreal	1	Diurnal	2	Yes (Akbar et al. 2019)	1	**4**
Trachypithecus auratus	Ebony leaf monkey	Arboreal	1	Diurnal	2	Yes	1	**4**
Presbytis thomasi	Thomas's leaf monkey	Arboreal	1	Diurnal	2	Yes	1	**4**
Presbytis melalophos	Sumatran leaf monkey	Arboreal	1	Diurnal	2	Yes	1	**4**
Symphalangus syndactylus	Siamang	Arboreal	1	Diurnal	2	Yes	1	**4**
Hylobates agilis	Agile gibbon	Arboreal	1	Diurnal	2	Yes (Buckley et al. 2006)	1	**4**
Hylobates lar	White-handed gibbon	Arboreal	1	Diurnal	2	Yes	1	**4**

Table S5. Calculations for **Processability: TEETH REMOVAL** scores of species observed in urban markets in Medan, North Sumatra, in 1997-2008 (Shepherd, 2010).

**Species** (Shepherd, 2010)	**Common Name** (Shepherd, 2010)	**Teeth Removed?** (Shepherd et al., 2004; Shepherd, 2010)	**TEETH REMOVAL / PROCESSABILITY SCORE**
Nycticebus coucang	Greater slow loris	Yes	**1**
Macaca fascicularis	Long-tailed macaque	No	**0**
Macaca nemestrina	Pig-tailed macaque	No	**0**
Trachypithecus cristatus	Silvered leaf monkey	No	**0**
Trachypithecus auratus	Ebony leaf monkey	No	**0**
Presbytis thomasi	Thomas's leaf monkey	No	**0**
Presbytis melalophos	Sumatran leaf monkey	No	**0**
Symphalangus syndactylus	Siamang	Yes	**1**
Hylobates agilis	Agile gibbon	Yes	**1**
Hylobates lar	White-handed gibbon	Yes	**1**

Table S6. Calculations for **Usability: SUCCESS IN CAPTIVITY** scores of species observed in urban markets in Medan, North Sumatra, in 1997-2008 (Shepherd, 2010).

**Species** (Shepherd, 2010)	**Common Name** (Shepherd, 2010)	**Trophic Guild (Galan-Acedo et al. 2019; IUCN, 2025)**	**DIET FLEXIBILITY SCORE** 1: restricted diet; 2: lower diet flexibility; 3: higher diet flexibility	**TEETH REMOVAL SCORE** 1: teeth removed (Shepherd et al., 2004; Shepherd, 2010)	**SUCCESS IN CAPTIVITY SCORE**
Nycticebus coucang	Greater slow loris	omnivore	3	0	**3**
Macaca fascicularis	Long-tailed macaque	frugivore	2	1	**3**
Macaca nemestrina	Pig-tailed macaque	frugivore	2	1	**3**
Trachypithecus cristatus	Silvered leaf monkey	folivore	2	1	**3**
Trachypithecus auratus	Ebony leaf monkey	folivore	2	1	**3**
Presbytis thomasi	Thomas's leaf monkey	folivore	2	1	**3**
Presbytis melalophos	Sumatran leaf monkey	folivore-frugivore	3	1	**4**
Symphalangus syndactylus	Siamang	folivore-frugivore	3	0	**3**
Hylobates agilis	Agile gibbon	frugivore	2	0	**2**

Hylobates lar	White-handed gibbon	Frugivore	2	0	**2**

Table S7. Calculations for **Usability: HAS MULTIPLE USES** scores of species observed in urban markets in Medan, North Sumatra, in 1997-2008 (Shepherd, 2010).

**Species** (Shepherd, 2010)	**Common Name** (Shepherd, 2010)	**Other uses (beyond pet/ companionship)** (Shepherd et al. 2004)	**MULTIPLE USE SCORE**
Nycticebus coucang	Greater slow loris	Some sold for medicinal use (treatment for asthma)	**1**
Macaca fascicularis	Long-tailed macaque	Traded for food	**1**
Macaca nemestrina	Pig-tailed macaque	Traded for food	**1**
Trachypithecus cristatus	Silvered leaf monkey	Traded for food	**1**
Trachypithecus auratus	Ebony leaf monkey	No mention of other uses	**0**
Presbytis thomasi	Thomas's leaf monkey	No mention of other uses	**0**
Presbytis melalophos	Sumatran leaf monkey	No mention of other uses	**0**
Symphalangus syndactylus	Siamang	No mention of other uses	**0**
Hylobates agilis	Agile gibbon	No mention of other uses	**0**
Hylobates lar	White-handed gibbon	No mention of other uses	**0**

Table S8. Calculations for **ENJOYABIILITY** scores of species observed in urban markets in Medan, North Sumatra, in 1997-2008 (Shepherd, 2010).

**Species** (Shepherd, 2010)	**Common Name** (Shepherd, 2010)	**Avg. Body Mass (kg)** (Galan-Acedo et al. 2019)	**AVG. BODY MASS SCORE** 1: large; 2: medium; 3: small	**Age Group Traded** (Shepherd et al. 2004; Nijman 2009; Nijman et al. 2017)	**AGE GROUP TRADED SCORE** 1: mostly adults; 2: equal adults/non-adult; 3: mostly non-adults	**ENJOYABILITY SCORE**
Nycticebus coucang	Greater slow loris	0.65	3	Equal adults/non-adults	2	**5**
Macaca fascicularis	Long-tailed macaque	4.03	3	Mostly non-adults	3	**6**
Macaca nemestrina	Pig-tailed macaque	8.89	2	Mostly non-adults	3	**5**
Trachypithecus cristatus	Silvered leaf monkey	6.25	2	Mostly non-adults	3	**5**
Trachypithecus auratus	Ebony leaf monkey	7.1	2	Mostly non-adults	3	**5**
Presbytis thomasi	Thomas's leaf monkey	6.73	2	Mostly non-adults	3	**5**

Presbytis melalophos	Sumatran leaf monkey	6.53	2	Mostly non-adults	3	**5**
Symphalangus syndactylus	Siamang	11.3	1	Mostly non-adults	3	**4**
Hylobates agilis	Agile gibbon	6	2	Mostly non-adults	3	**5**
Hylobates lar	White-handed gibbon	5.64	2	Mostly non-adults	3	**5**

Table S9. Calculations for **Value/Desirability: HOLDS ECOLOGICAL VALUE** scores of species observed in urban markets in Medan, North Sumatra, in 1997-2008 (Shepherd, 2010).

**Species** (Shepherd, 2010)	**Common Name** (Shepherd, 2010)	**Other uses (beyond pet/ companionship)** (Shepherd et al. 2004)	**ECOLOGICAL VALUE SCORE**
Nycticebus coucang	Greater slow loris	Some sold for medicinal use (meat, treatment for asthma)	**1**
Macaca fascicularis	Long-tailed macaque	Traded for food	**1**
Macaca nemestrina	Pig-tailed macaque	Traded for food	**1**
Trachypithecus cristatus	Silvered leaf monkey	Traded for food	**1**
Trachypithecus auratus	Ebony leaf monkey	No mention of ecological value	**0**
Presbytis thomasi	Thomas's leaf monkey	No mention of ecological value	**0**
Presbytis melalophos	Sumatran leaf monkey	No mention of ecological value	**0**
Symphalangus syndactylus	Siamang	No mention of ecological value	**0**
Hylobates agilis	Agile gibbon	No mention of ecological value	**0**
Hylobates lar	White-handed gibbon	No mention of ecological value	**0**

Table S10. Calculations for **Value/Desirability: CONSERVATION VALUE/RARITY** scores of species observed in urban markets in Medan, North Sumatra, in 1997-2008 (Shepherd, 2010).

**Species** (Shepherd, 2010)	**Common Name** (Shepherd, 2010)	**IUCN Threat Status** (Shepherd, 2010)	**IUCN THREAT/ RARITY SCORE** 1: LC; 2: NT; 3: VU; 4: EN; 5: CR	**Endemic to Indonesia?** (IUCN, 2025) 1: Yes	**CONSERVATION VALUE SCORE**
Nycticebus coucang	Greater slow loris	Vulnerable (VU)	3	0	**3**
Macaca fascicularis	Long-tailed macaque	Least Concern (LC)	1	0	**1**
Macaca nemestrina	Pig-tailed macaque	Vulnerable (VU)	3	0	**3**
Trachypithecus cristatus	Silvered leaf monkey	Near Threatened (NT)	2	0	**2**

Trachypithecus auratus	Ebony leaf monkey	Vulnerable (VU)	3	1	**4**
Presbytis thomasi	Thomas's leaf monkey	Vulnerable (VU)	3	1	**4**
Presbytis melalophos	Sumatran leaf monkey	Endangered (EN)	4	1	**5**
Symphalangus syndactylus	Siamang	Endangered (EN)	4	0	**4**
Hylobates agilis	Agile gibbon	Endangered (EN)	4	0	**4**
Hylobates lar	White-handed gibbon	Endangered (EN)	4	0	**4**

Table S11. Calculations for **Value/Desirability: HOLDS SYMBOLIC VALUE** scores of species observed in urban markets in Medan, North Sumatra, in 1997-2008 (Shepherd, 2010).

**Species** (Shepherd, 2010)	**Common Name** (Shepherd, 2010)	**Symbolic role** (Shepherd et al. 2004)	**SYMBOLIC VALUE SCORE**
Nycticebus coucang	Greater slow loris	No mention of symbolic roles	**0**
Macaca fascicularis	Long-tailed macaque	No mention of symbolic roles	**0**
Macaca nemestrina	Pig-tailed macaque	No mention of symbolic roles	**0**
Trachypithecus cristatus	Silvered leaf monkey	Considered a 'novelty' species in the markets	**1**
Trachypithecus auratus	Ebony leaf monkey	Considered a 'novelty' species in the markets	**1**
Presbytis thomasi	Thomas's leaf monkey	Considered a 'novelty' species in the markets	**1**
Presbytis melalophos	Sumatran leaf monkey	Considered a 'novelty' species in the markets	**1**
Symphalangus syndactylus	Siamang	No mention of symbolic roles	**0**
Hylobates agilis	Agile gibbon	Considered a 'novelty' species in the markets	**1**
Hylobates lar	White-handed gibbon	Considered a 'novelty' species in the markets	**1**

Figure S1. Variable Correlation Circle from principal component regression to analyze the relationship between conservation criminology variables to assess primate species vulnerability to illicit trade and number of individual primates detected in market surveys for 10 species in Medan, North Sumatra (1997-2008; Shepherd, 2010). D = demand-side variable, S = supply-side variable.

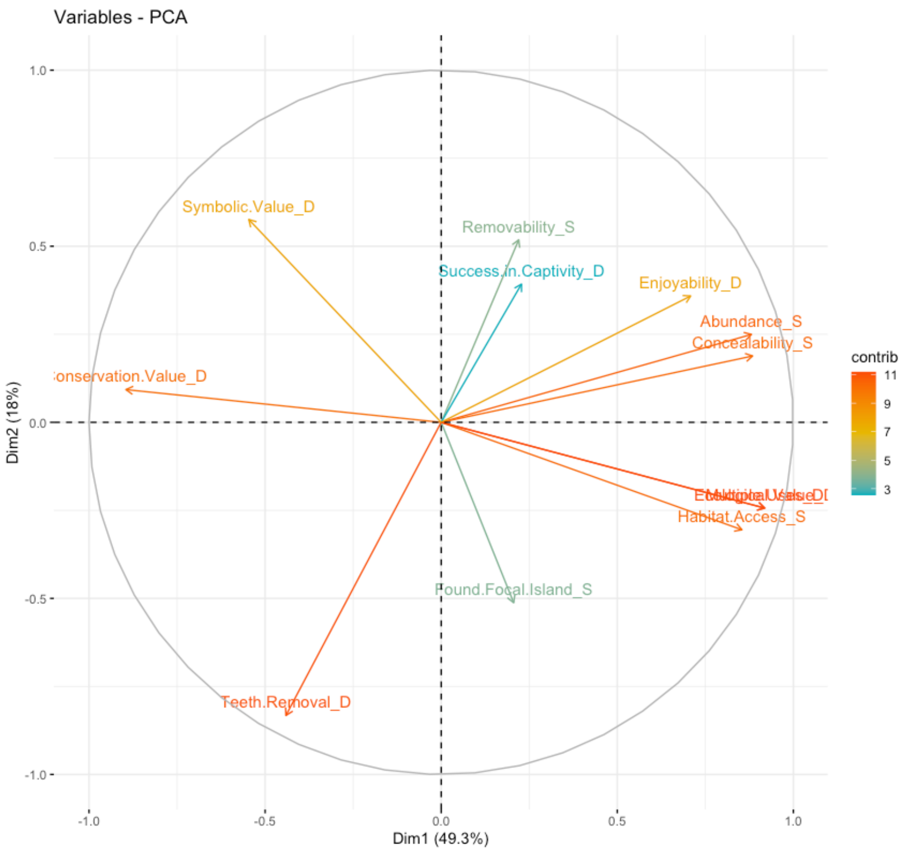

SUPPLEMENTARY INFORMATION REFERENCES

Akbar, M. A., Rizaldi, R., Novarino, W., Perwitasari-Farajallah, D., & Tsuji, Y. (2019). Activity budget and diet in silvery lutung Trachypithecus cristatus at Gunung Padang, West Sumatra, Indonesia. *Biodiversitas Journal of Biological Diversity*, *20*(3), 719-724.

Buckley, C., Nekaris, K. A. I., & Husson, S. J. (2006). Survey of Hylobates agilis albibarbis in a logged peat-swamp forest: Sabangau catchment, Central Kalimantan. *Primates*, *47*, 327-335.

Galán-Acedo, C., Arroyo-Rodríguez, V., Andresen, E., & Arasa-Gisbert, R. (2019). Ecological traits of the world's primates. *Scientific Data*, *6*(1), 55.

IUCN. (2025). International union for conservation of nature, IUCN Red List of Threatened Species. Version 2021-3. www.iucnredlist.org

Nijman, V., Spaan, D., Rode-Margono, E. J., Wirdateti, and Nekaris, K. A. I. (2017). Changes in the primate trade in Indonesian wildlife markets over a 25-year period: Fewer apes and langurs, more macaques, and slow lorises. *Ame. J. Primatol.* 79. doi: 10.1002/ajp.22517

Shepherd, C. R., Sukumaran, J., and Wich, S. A. (2004). Open season: an analysis of the pet trade in Medan, Sumatra 1997-2001. Petaling Jaya, Malaysia: TRAFFIC Southeast Asia.

Shepherd, C. (2010). Illegal primate trade in Indonesia exemplified by surveys carried out over a decade in North Sumatra. *Endanger. Species Res.* 11, 201-205.
